# Overcoming Diagnostic Challenges: A Rare Presentation of Primary Hemophagocytic Lymphohistiocytosis (HLH) in a Young Female and the Importance of Timely Recognition

**DOI:** 10.7759/cureus.60242

**Published:** 2024-05-13

**Authors:** Karthiga Selvaratnam, MSM Nusair, Pakkiyaretnam Mayurathan

**Affiliations:** 1 Internal Medicine Department, Teaching Hospital Batticaloa, Batticaloa, LKA; 2 University Medical Unit, Teaching Hospital Batticaloa, Batticaloa, LKA; 3 Clinical Sciences Department, Faculty of Health-Care Sciences, Eastern University Sri Lanka, Batticaloa, LKA

**Keywords:** hemophagocytic lymphohistiocytosis (hlh), secondary hlh, unc13d gene, prf1 gene, familial hlh, cytokine storm, etoposide, abnormal immune activation

## Abstract

Hemophagocytic lymphohistiocytosis (HLH) is a severe life-threatening hematological disorder characterized by the dysregulation of the immune system and a hyperinflammatory response. Prompt treatment is crucial to prevent fatality. Although primarily affecting infants, HLH can also occur in children and adults. It is classified as primary and secondary, with primary HLH being genetic and predominantly affecting children. Secondary HLH is triggered by infections, malignancy, metabolic disorders, and rheumatological conditions. Diagnosis is based on the HLH-2004 criteria, considering clinical and laboratory parameters. Early diagnosis and treatment improve prognosis. Treatment follows the HLH-94 and HLH-2004 protocol and consists of eight weeks of induction therapy with cyclosporine, corticosteroids, and etoposide. This case describes a 26-year-old female diagnosed with HLH and successfully treated according to the protocol. The patient exhibited improvement and was discharged, demonstrating the importance of early diagnosis and appropriate management in adult HLH cases.

## Introduction

Hemophagocytic lymphohistiocytosis (HLH) is a rapidly progressive hyperinflammatory condition characterized by abnormal immune activation and cytokine storm, leading to multiorgan dysfunction and a potentially fatal outcome. The incidence of HLH is approximately 1.2 in 1,000,000 individuals per year worldwide [1]. HLH can be classified as primary or familial, associated with autosomal recessive inheritance and mutations in genes such as *PRF1* or *UNC13D*, which account for 40%-60% of familial HLH cases [2,3]. Secondary or acquired HLH is typically triggered by infections, malignancy, metabolic disorders, and autoimmune/rheumatological conditions.

Infection is a common trigger for both familial and acquired HLH, with Epstein-Barr virus (EBV) being the most frequently associated pathogen [3]. Etoposide therapy has shown effectiveness in treating EBV-induced HLH [4]. The clinical features of both primary and secondary HLH often overlap and resemble sepsis syndrome [5]. Common manifestations include prolonged fever, splenomegaly, pancytopenia, elevated transaminases, and high ferritin levels.

The HLH-2004 criteria are used to diagnose HLH, requiring the fulfillment of five out of nine specific criteria. The Hscore is another diagnostic tool used to assess reactive HLH [6]. Early diagnosis is critical in managing HLH and necessitates a high level of clinical suspicion.

## Case presentation

In this case, a 26-year-old female presented to the primary care unit with a history of intermittent fever of about three weeks. Fever was associated with lethargy, tiredness, cough with sputum, loss of appetite, and weight loss. She did not exhibit symptoms such as sore throat, night sweats, or hemoptysis, and she had no contact history of tuberculosis. There was no chest pain, shortness of breath, headache, or dizziness. She did not have any symptoms or signs suggestive of meningeal irritation such as severe headache, altered mental status, Kernig sign, or Brudzinski sign. The patient denied nausea, vomiting, abdominal pain, altered bowel habits, dysuria, hematuria, or increased urinary frequency. Her urine output was normal.

There was no history of joint pain, swelling, oral ulcers, hair loss, or rashes, and there were no bleeding manifestations. The patient denied recent travel, contact with animals, long-term drug use, high-risk sexual behavior, blood transfusion, or substance abuse in the past. She had three previous hospital admissions within the last two months with similar presentations. The first one was managed as dengue hemorrhagic fever with dengue viral hepatitis at a local hospital even though dengue serological investigation was negative, and the subsequent admission was treated as sepsis. Persistent pancytopenia with high transaminase levels was noted in all three admissions. Her menstrual and obstetric history were unremarkable, and she is a mother of a 10-month-old child.

On admission, the patient appeared drowsy, ill-looking, and pale. She had mild dyspnea and tachycardia (pulse rate {PR}: 110/minute) with a stable blood pressure of 110/70 mmHg. Oxygen saturation (SpO_2_) at room air was 90%. There were no signs of neck stiffness, lymphadenopathy, features of infective endocarditis, or autoimmune diseases. There were no tattoo marks or intravenous (IV) injection sites observed. Bilateral crepitation in the lung bases and mild hepatomegaly with moderate splenomegaly were noted during the abdominal examination. The rest of the physical examinations were normal.

**Figure 1:**
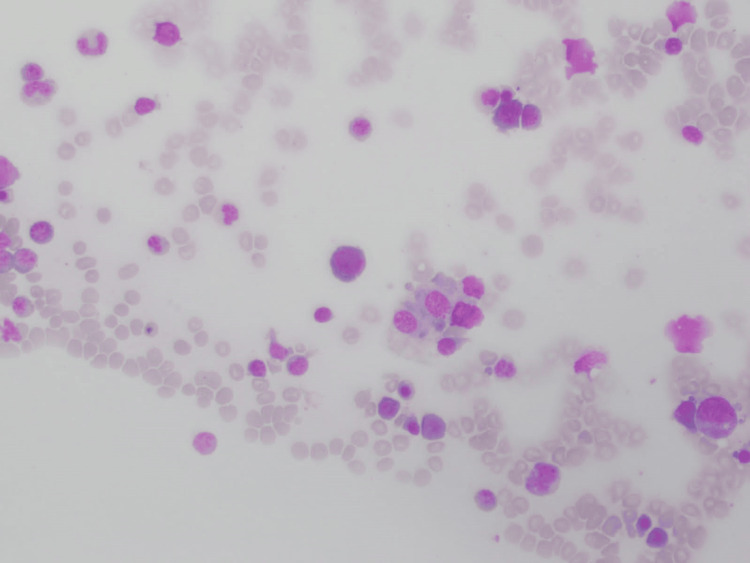
Bone marrow trephine biopsy showing hemophagocytosis

Laboratory investigations revealed pancytopenia (WBC, 1.37×10^3^/µL; neutrophils, 53.3%; lymphocytes, 43.1%; hemoglobin {Hb}, 5.2 g/dL; and platelet, 50,000/µL). Liver function tests showed increased transaminase levels (aspartate aminotransferase {AST}, 767 U/L; alanine aminotransferase {ALT}, 518 U/L) along with hyperferritinemia (84,000 ng/mL) and hypertriglyceridemia (456 mg/dL). Plasma fibrinogen level was normal (240 mg/dL), but the rotational thromboelastometry (ROTEM) study indicated mild fibrinogen deficiency. Lactate dehydrogenase (LDH) was elevated (1,689 U/L), C-reactive protein (CRP) levels were high (57 mg/L), and erythrocyte sedimentation rate (ESR) was within the normal range (12 mm/hour). Bone marrow aspirate and trephine biopsy showed evidence of hemophagocytosis (Figure 1).

**Figure 2:**
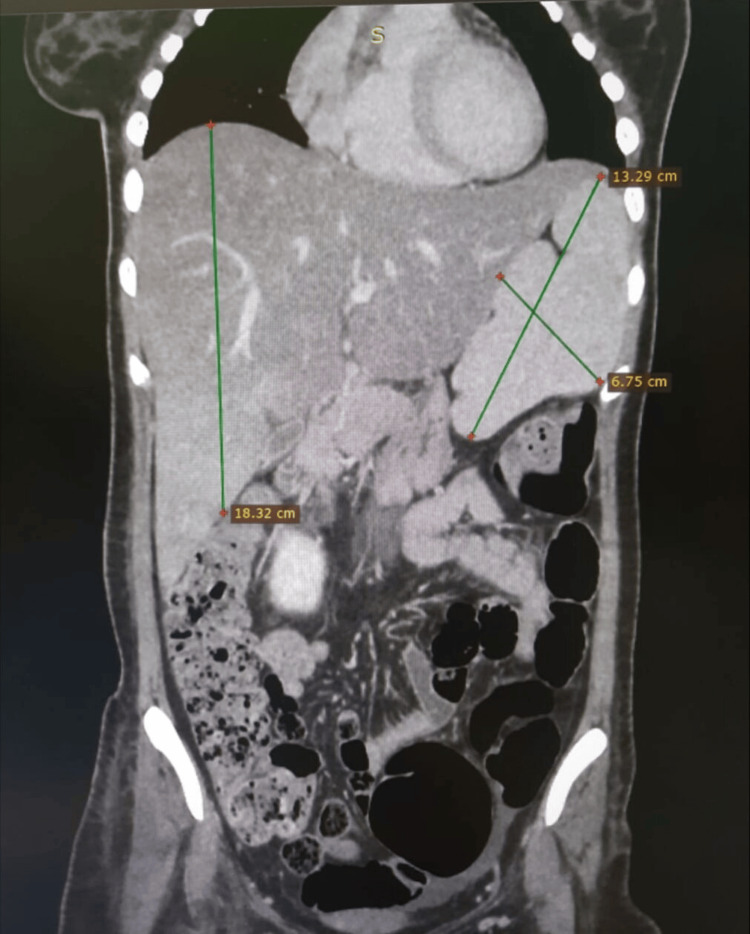
Contrast-enhanced CT of the abdomen showing moderate hepatomegaly (18.3 cm in size) and splenomegaly (13.29×6.75 cm in size) CT: computed tomography

**Figure 3:**
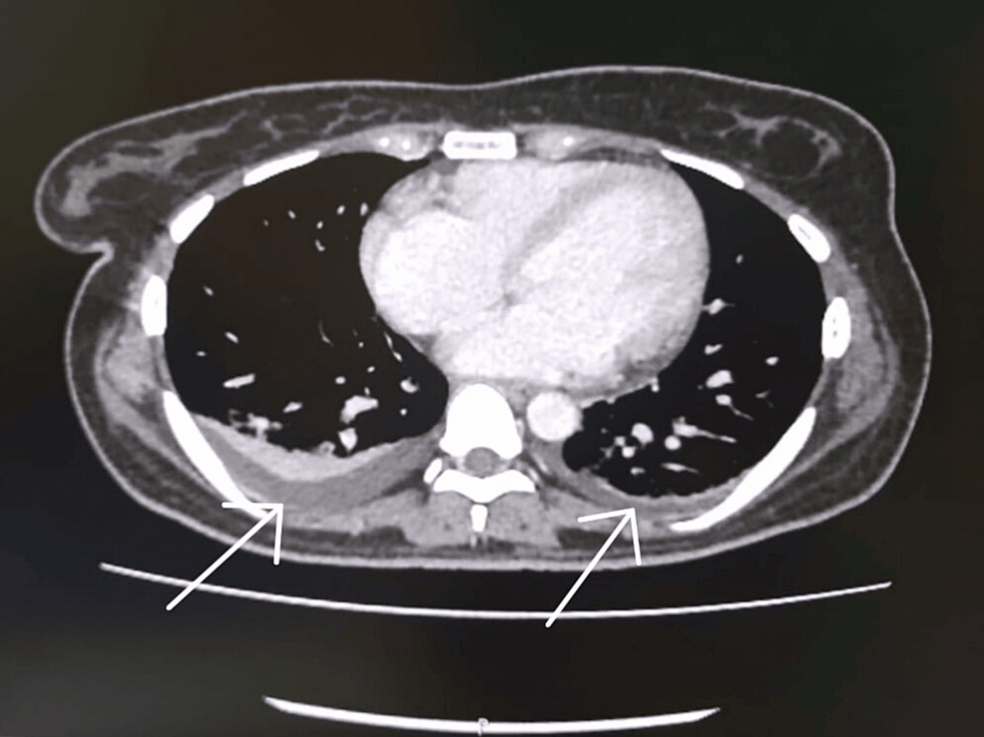
Contrast-enhanced CT of the chest showing bilateral pleural effusion with adjacent collapse consolidation (white arrows) No lung masses, nodules, or cavitatory lesions. No hilar or mediastinal lymphadenopathy CT: computed tomography

An ultrasound of the abdomen revealed splenomegaly, hepatomegaly, and fatty liver changes. Chest X-ray and two-dimensional (2D) echocardiogram were unremarkable. Further imaging with a contrast-enhanced computed tomography (CECT) of the abdomen and chest revealed moderate hepatosplenomegaly (Figure 2) and bilateral pleural effusion with adjacent collapse consolidation (Figure 3) of the lung. There are no lung masses, nodules, cavitatory lesions, or hilar lymphadenopathy.

A liver biopsy indicated moderate lymphocytic steatohepatitis with multiple foci of hepatic necrosis. Investigations were conducted to exclude secondary causes of HLH, and all results were negative (Table 1).

**Table 1:** Investigations to exclude secondary causes ANA, antinuclear antibody; NS1, nonstructural protein 1; IgM, immunoglobulin M; IgG, immunoglobulin G; AFB, acid-fast bacilli; TB, tuberculosis; PCR, polymerase chain reaction; EBV, Epstein-Barr virus; IFA, immunofluorescent assay; TSH, thyroid-stimulating hormone; FT4, free thyroxine

Investigations	Results
Malaria parasite	Negative
ANA	Negative
Dengue NS1	Negative
Dengue IgM and IgG	Negative
Sputum AFB and TB PCR	Negative
Serology for hepatitis B	Negative
Serology for hepatitis C	Negative
Serology for HIV	Negative
EBV antibody	Negative
*Cytomegalovirus* antibody	Negative
*Rickettsia* antibody/IFA	Negative
*Brucella* PCR	Negative
Urine/blood cultures	No growth
TSH	0.015 mIU/L
FT4	40.4 pmol/ L

On admission, she was very ill, and as the patient's SpO_2_ was 90%, supplemental oxygen was given. With 5 L of oxygen via face mask, her SPO_2_ increased to 98% (arterial blood gas {ABG} pH, 7.51; partial pressure of carbon dioxide (pCO_2_), 24.9 mmHg; partial pressure of oxygen {pO_2_}, 88.4 mmHg; and bicarbonate {HCO_3_}, 20.1 mmol/L). Broad-spectrum antibiotics were started. On the second day of admission, the patient was transferred to the intensive care unit. Based on clinical and laboratory results, the diagnosis of HLH was made on the third day. Six out of eight HLH-2004 criteria were fulfilled (fever of >38.5°C, splenomegaly, pancytopenia, hypertriglyceridemia, hyperferritinemia, and hemophagocytosis in the bone marrow). After excluding all possible secondary causes of HLH, the patient was diagnosed with primary HLH. Unfortunately, the genetic panel for HLH could not be performed due to a lack of facilities at our hospital.

An IV methylprednisolone 500 mg daily along with granulocyte colony-stimulating factor at a dose of 300 μg was given for three days. As there was no significant improvement with the above management, a two-day course of IV immunoglobulin was also given at a dose of 1 mg/kg/day. The HLH-2004 protocol, as well as the neutropenic prophylactic protocol, was initiated simultaneously following a multidisciplinary team approach.

IV dexamethasone was started at a dose of 10 mg/m²/day, and oral cyclosporine was initiated at a dose of 100 mg daily. IV etoposide was started at a dose of 150 mg/m² twice a week for the first two weeks and once weekly for six weeks. The patient showed dramatic clinical and biochemical improvement after the initiation of HLH-specific therapy. The patient was discharged after completing the eighth dose of etoposide therapy with oral cyclosporine and dexamethasone. On discharge, all the patient's biochemical parameters were normal. In the outpatient clinic follow-up, an oral cyclosporine tail-off regime (100 mg mane and 50 mg noct for one week, 50 mg twice daily for one week, and 50 mg daily for one week and then stop) with low-dose oral dexamethasone (1 mg daily for three days and 0.5 mg daily for three days and then stop) was started. After one month of clinic follow-up, all medications were stopped, and the patient was doing well.

## Discussion

HLH is an uncommon deadly hematological disease defined by excess cytokine response and a hyperinflammatory response. The rapid diagnosis of HLH is important for initiating specific therapy, as delay in diagnosis can lead to poor outcomes. However, HLH is often misdiagnosed in adults due to its rarity and varying clinical characteristics. HLH can occur as either primary (familial) or secondary (acquired).

Our patient was initially treated as dengue hemorrhagic fever and sepsis and then presented with a prolonged fever of 20 days at our primary care unit. On the day of admission, we considered HLH as one of the differential diagnoses. The diagnosis of HLH was made using the HLH-2004 diagnostic criteria (Table 2) [7], and our patient fulfilled six of the eight criteria.

**Table 2:** HLH diagnostic criteria HLH, hemophagocytic lymphohistiocytosis; NK, natural killer; CD25, cluster of differentiation 25

HLH diagnostic criteria
Fever (temperature of >38.5°C)
Splenomegaly
Cytopenia in peripheral blood film (at least two of three cell lineages)
High triglyceride (>265 mg/dL) and/or low fibrinogen (<150 mg/dL)
Hemophagocytosis in the bone marrow, spleen, lymph node, or liver
Low or absent NK cell activity
Hyperferritinemia of >500 ng/mL
High-soluble CD25

In our case, natural killer (NK) cell activity and cluster of differentiation 25 (CD25) level were not performed due to unavailability.

Although hemophagocytosis is present in the bone marrow biopsy, it is not a pathognomonic feature of HLH and is not required for diagnosis [7]. However, hyperferritinemia of >10,000 µg/L is a sensitivity of 90% and a specificity of 96% for the diagnosis of HLH [8]. The Hscore is a new criterion used to improve the diagnosis of HLH, with a cutoff value of 168 revealing 100% and 94.1% sensitivity and specificity, respectively [9]. In our case, the Hscore is 269, indicating a 99% probability of HLH.

Lung involvement in HLH is rare and carries a poor prognosis. According to a retrospective study, 54% of patients with HLH had lung involvement, which includes radiological evidence of centrilobular nodules, consolidation, ground glass opacities, pleural effusion, and mediastinal lymphadenopathy [10]. Our patient had bilateral pleural effusion with adjacent collapse and consolidation.

We did not perform a genetic test on our patient because the test was unavailable in Sri Lanka. Therefore, we could not rule out the possibility of primary HLH. However, initially, our patient was managed as dengue hemorrhagic fever, but dengue immunoglobulin G (IgG) and immunoglobulin M (IgM) were negative. Extensive studies were conducted to identify secondary causes, but no etiology for HLH was identified, including malignancy, infections, and metabolic or autoimmune diseases. In the majority of cases, secondary causes of HLH have not been identified. Late-onset HLH in which the etiology is not identified may be due to primary HLH [11].

Although the absence of family history is against the possibility of primary HLH in our case, it should be noted that primary cases may not always have a family history due to autosomal recessive inheritance [12].

Early diagnosis is a crucial step in the management of HLH, requiring a high degree of clinical suspicion and a series of investigations. Prompt therapy following diagnosis can be lifesaving. If a patient is acutely ill, HLH-specific therapy based on HLH-94/HLH-2004 protocols should be initiated. If the patient is hemodynamically stable, HLH could be treated with corticosteroids before starting chemotherapy. Allogeneic hematopoietic cell transplantation is mainly reserved for patients with refractory cases, familial HLH, central nervous system (CNS) involvement, and underlying malignancy [13].

The prognosis of HLH is generally poor if left untreated. The median survival of patients treated with the HLH-94 protocol is 54% at 6.2 years [13,14]. Several factors can influence the prognosis in adult patients, including high ferritin levels, advanced age, underlying malignancy, and elevated levels of AST and LDH.

In our case, the patient showed a positive response to HLH-2004 therapy, which included the administration of dexamethasone, etoposide, and cyclosporine. This treatment approach has been effective in managing HLH and improving patient outcomes.

Regular follow-up and monitoring are crucial in managing HLH to ensure that the disease remains under control and to detect any potential relapses or complications. The patient's positive response to treatment and ongoing care from the clinic are positive indicators for her long-term prognosis.

## Conclusions

HLH is an aggressive rare clinical disorder. Primary HLH is common among children when compared to adults. Anyone with ongoing fever, pancytopenia, and organomegaly should be suspected of HLH. It may be fatal if early diagnosis and treatment are not initiated.
